# Application of Humanized MHC Transgenic Mice in the Screening of HLA–Restricted T Cell Epitopes for Influenza Vaccines

**DOI:** 10.3390/vaccines13030331

**Published:** 2025-03-20

**Authors:** Yuwei Wei, Keyu Sun, Xuelian Han, Yali Sun, Jiejie Zhang, Yuan Wang, Qi Yin, Tiantian Yang, Kai Yuan, Min Li, Guangyu Zhao

**Affiliations:** 1 State Key Laboratory of Pathogen and Biosecurity, Academy of Military Medical Sciences, Beijing 100071, China; weiyuwei9872@163.com (Y.W.); hanxuelian@bmi.ac.cn (X.H.); zhangjiejie202202@163.com (J.Z.); wangyuan1@bmi.ac.cn (Y.W.); yinqi@bmi.ac.cn (Q.Y.); 2 Public Health School, Mudanjiang Medical University, Mudanjiang 157011, China; s1010457183@163.com (K.S.); 18852551076@163.com (Y.S.); ytt_tjutcm@163.com (T.Y.); ykmarceau@163.com (K.Y.); 3 School of Basic Medical Sciences, Anhui Medical University, Hefei 230032, China

**Keywords:** influenza A virus, mouse model, human leukocyte antigen (HLA) complex, HLA-restricted T cell epitope

## Abstract

Background: Annual influenza epidemics pose a significant burden on the global healthcare system. The currently available vaccines mainly induce the production of neutralizing antibodies against hemagglutinin and neuraminidase, which are prone to antigenic variation, and this can reduce vaccine efficacy. Vaccines designed to target T cell epitopes can be potentially valuable. Considering the difficulties in obtaining clinical samples and the unique advantages of mice in disease-related research, a mouse model that can simulate human immune responses can be a superior alternative to peripheral blood mononuclear cells for epitope screening. Methods: The T cell epitopes of the A/California/07/2009 (H1N1) virus were predicted and utilized to evaluate the cellular immune responses of HLA-A2/DR1 and HLA-A11/DR1 transgenic mice during epitope screening. The selected peptides were used to immunize these two groups of transgenic mice, followed by a viral challenge to assess their protective efficacy. Results: The epitopes that were predicted and screened could stimulate cellular immune responses in HLA-A2/DR1 transgenic mice, HLA-A11/DR1 transgenic mice, and C57BL/6 mice. Moreover, the transgenic mice exhibited stronger ability to produce IFN-γ than that of the wild-type mice. Upon immunization and subjecting to viral challenge, the selected peptides exhibited protective effects against the influenza virus. Conclusions: The HLA-A2/DR1 and HLA-A11/DR1 transgenic mouse models can be used for the direct screening and validation of influenza virus T cell epitopes, which is crucial for designing T cell epitope vaccines against influenza viruses. Further, this method can be applied in epitope screening and vaccine designing before the spread of other emerging and sudden infectious diseases, thereby supporting epidemic control.

## 1. Introduction

The influenza virus, which belongs to the *Orthomyxoviridae* family, is a negative-sense, single-stranded RNA virus with a segmented genome [1,2]. Influenza A virus (IAV), influenza B virus, and influenza C virus can infect humans. Among these, IAV is the most prevalent, and it has caused multiple influenza pandemics in history [2,3]. According to an epidemiological study conducted by the World Health Organization (WHO), seasonal influenza epidemics result in 3–5 million severe illnesses and 290,000–650,000 deaths worldwide each year [4], posing a significant burden on the global healthcare systems.

After the host is infected with the virus, the humoral immune response is mediated by B cells. Activated B cells differentiate into plasma cells, which secrete large amounts of neutralizing antibodies. These antibodies can bind to viral surface proteins and prevent the virus from interacting with host cell receptors [5]. Cytotoxic T cells contribute to viral clearance by eliminating virus-infected cells [6], while neutralizing antibodies plays a key role in targeting extracellular virus particles. According to the global action plan of the WHO, annual vaccination is the best method to control the medical, social, and economic burden caused by influenza [7]. Currently available vaccines include subunit vaccines, live attenuated vaccines, and inactivated surface protein vaccines [8]. Most of these vaccines mainly induce neutralizing antibody responses against the surface hemagglutinin (HA) glycoprotein. However, the HA glycoprotein of influenza viruses is highly mutable, so vaccines from the previous year may not offer cross-protection against strains circulating in the current year [9,10]. Among the population infected with the influenza virus in 2009, CD8^+^ T cells specific to conserved viral epitopes correlated with cross-protection against symptomatic influenza [11]. Other studies have also shown that individuals with cross-reactive memory CD8^+^ T cells recover more quickly than those without memory CD8^+^ T cells [12,13]. Based on this finding, influenza vaccines targeting CD8^+^ T cell epitopes can be potentially effective in controlling the disease.

To screen and validate T cell epitopes, it is typically necessary to use peripheral blood mononuclear cells (PBMCs) extracted from clinical samples [14]. However, obtaining adequate PBMC samples from both newly infected and convalescent individuals is often challenging. Further, the human-expressed MHC molecules are extremely complex. Therefore, identifying easily accessible and quality-controlled alternative tools for screening T cell epitopes is necessary. From an experimental perspective, mice are small and inexpensive, can rapidly reproduce, and can be genetically manipulated. Thus, they are widely used in the field of disease-related research [15,16]. Therefore, a mouse model that can mimic the human immune response may be a good tool for designing CD8^+^ T cell epitope influenza vaccines.

The MHC in humans is known as the human leukocyte antigen (HLA) [17]. Based on a previous study, HLA-A2/DR1 (*HLA-A2^+/+^/DR01^+/+^/H-2-β2m^−/−^/IAβ^−/−^*) and HLA-A11/DR1 (*HLA-A11^+/+^/DR01^+/+^/H-2-β2m^−/−^/IAβ^−/−^*) transgenic mice can simulate human immune responses [18]. This study aimed to evaluate the suitability of these two mouse models in influenza epitope screening. Immunoinformatics tools were utilized to predict the T cell epitopes of the A/California/07/2009 (H1N1) influenza virus. Subsequently, the humanized MHC mouse models, HLA-A2/DR1 transgenic mice and HLA-A11/DR1 transgenic mice, were used for epitope screening. The mice were immunized with the selected epitopes and subjected to viral challenge to validate the protective efficacy of the epitopes. Results showed that the epitopes screened using both HLA-A2/DR1 and HLA-A11/DR1 transgenic mice exhibited protective effects. Therefore, these two humanized MHC mouse models can effectively screen the T cell epitopes of the influenza virus.

## 2. Materials and Methods

### 2.1. Sequence Retrieval of the A/California/07/2009 (H1N1) Genes

Reference sequences of the eight gene segments of A/California/07/2009 (H1N1) were obtained from the National Center for Biotechnology Information database (https://www.ncbi.nlm.nih.gov/ (accessed on 27 September 2024)) in the FASTA format.

### 2.2. Bioinformatic Prediction Strategy for T Cell Epitopes Derived from the IAV

HLA-A*02:01- and HLA-A*11:01-restricted T cell epitopes were predicted using the online T cell epitope prediction tools from the Immune Epitope Database (https://www.iedb.org/ (accessed on 30 September 2024)), EpiJen (https://www.ddg-pharmfac.net/epijen/EpiJen/EpiJen.htm (accessed on 30 September 2024)), and SYFPEITHI (http://www.syfpeithi.de/bin/MHCServer.dll/EpitopePrediction.htm (accessed on 30 September 2024)). In the Immune Epitope Database T cell epitope prediction tool, three prediction methods were selected: NetMHCPan 4.1 EL, ANN 4.0, and SMM.

### 2.3. Peptides

The peptide was synthesized by HKGCBIO (Beijing) Co., Ltd., Beijing, China, with a purity of ≥98%. The peptide was aliquoted at a concentration of 1 mg per vial and stored frozen at −80 °C. The peptide was dissolved before use.

### 2.4. Mice and Ethical Approval

C57BL/6 mice (female, 6–8 weeks of age) were purchased from SPF Biotechnology Co., Ltd. (Beijing, China). Further, 6–8-week-old female HLA-A2/DR1 (HLA-A2^+/+^/DR01^+/+^/H-2-β2m^−/−^/IAβ^−/−^) transgenic mice and HLA-A11/DR1 (HLA-A11^+/+^/DR01^+/+^/H-2-β2m^−/−^/IAβ^−/−^) transgenic mice were obtained from the State Key Laboratory of Pathogen and Biosecurity. The mice were housed in specific pathogen-free animal experimental-barrier systems. The Institutional Animal Care and Use Committee of the Academy of Military Medical Sciences (permit number: IACUC-IME-2022-004) reviewed and approved all animal experimental protocols.

### 2.5. Influenza Virus Infection

The mice were divided into the HLA-A2/DR1, HLA-A11/DR1, and C57BL/6 groups, with 10 mice in each group. Each major group was then randomly divided into two subgroups, with five mice in each subgroup. In total, 30 mice were anesthetized with isoflurane, and baseline serum samples were collected. Then, all the mice were infected intranasally with 10^1.5^ TCID_50_ of the A/California/07/2009 (H1N1) influenza virus. To ensure mice survive until sampling, this dosage was set much lower than the lethal dosage [19,20]. In each major group, the first subgroup was euthanized on day 15 postinfection, and serum and spleen samples were collected. The second subgroup was euthanized on day 27 postinfection, and the spleens were collected. The serum was used to detect antibodies, and the spleens were used for single-cell preparation and the ELISpot assays. All experiments involving live influenza virus were conducted in a biosafety level-2 containment facility.

### 2.6. Spleen Single-Cell Preparation

The mice were euthanized via cervical dislocation, and the spleens were harvested under sterile conditions. The spleens were then placed in 6-well plates containing 3 mL of RPMI 1640 medium with 2% FBS. The spleens were gently homogenized using a grinder and a 70 μm cell strainer to obtain a single-cell suspension. After centrifugation at 300× *g* for 5 min at 4 °C, cells were resuspended in ammonium–chloride–potassium lysis buffer for 2 min. Lysis was stopped by adding RPMI 1640 medium with 2% FBS, and the cells were then centrifuged. Finally, the cells were resuspended in 2 mL of RPMI 1640 medium containing 2% FBS and diluted to a concentration of 5 × 10^5^ cells/mL.

### 2.7. ELISpot Assay

The ELISpot plates (Mabtech, Kista, Sweden), pre-coated with the mouse interferon-gamma (IFN-γ) antibody, were washed four times with sterile PBS, and each well was blocked with 200 μL of RPMI 1640 medium containing 10% FBS at room temperature for 2 h. After blocking, the plates were washed again four times with sterile PBS. The synthesized peptides were used to stimulate splenocytes from different strains of mice, with each well receiving 0.4 μg of the corresponding peptide and three replicates for each peptide. The Ebola virus epitope peptides were used as irrelevant negative controls, with 0.4 μg of the corresponding peptide per well. The positive control group received 100 μL of Concanavalin A (ConA, 2.5 μg/mL) per well. Meanwhile, the unstimulated group and the blank control group received 100 μL of RPMI 1640 medium per well. Except for the blank control group, 5 × 10^5^ prepared mouse spleen cells were added to each well. The blank control group wells contained 100 μL of RPMI 1640 medium. Subsequently, the plates were incubated at 37 °C in a CO_2_ incubator (5%) for 18–24 h. After incubation, the culture medium was discarded, and the plates were washed five times with PBS, incubating for 3–5 min during each wash. R4-6A2 biotinylated detection antibody (100 μL) was added to each well, followed by incubation at room temperature for 2 h. The plates were then washed five times with PBS, incubating for 3–5 min during each wash. Next, 100 μL of Streptavidin (HRP) was added to each well, followed by incubation at room temperature for 1 h. The plates were washed again five times with PBS, incubating for 3–5 min during each wash. The 3,3′,5,5′-tetramethylbenzidine substrate solution was added to each well (100 μL), and the plates were incubated at room temperature in the dark for 2–15 min. Then, the liquid inside the plates was discarded, and deionized water was used to slowly wash the plates 2–3 times to stop the color reaction. Finally, the ELISpot plates were inverted and air-dried at room temperature in the dark for more than 1 day before counting.

### 2.8. Antibody Detection

The specific antibodies in the serum of the mice were detected using the hemagglutination inhibition (HI) test, enzyme-linked immunosorbent assay (ELISA), and serum virus neutralization.

For the HI test, an equal volume of 5% turkey red blood cells was initially added to the serum, and the samples were incubated at room temperature for 30 min to eliminate nonspecific agglutination from the serum. Then, samples were centrifuged at 14,000× *g* for 15 s. Next, the supernatants were collected, and three replicate wells were set up for each sample and subjected to a two-fold serial dilution in hemagglutination plates. Four hemagglutination units of virus solution were added to the plates, and the plates were incubated at room temperature for 10 min. After incubation, 1% chicken red blood cells were added, and the plates were incubated at room temperature for 30 min. Next, the development of agglutination in the hemagglutination plates was evaluated. The highest antibody dilution that caused the inhibition of hemagglutination was identified as the HI titer.

In the ELISA plates (JetBio-Fil, Guangzhou, China), each well was coated with 0.5 μg of hemagglutinin protein of A/California/07/2009 (H1N1) and incubated overnight at 4 °C. After incubation, the plates were washed with PBST and then blocked with PBS containing 2% BSA at 37 °C for 2 h. After washing the plates, two-fold serially diluted serum was added, with three replicates for each sample, and incubated at 37 °C for 1 h. After washing the plates again with PBST, the HRP-conjugated secondary antibodies (Beyotime, Shanghai, China) were used to detect antigen-specific serum antibodies at 37 °C for 30 min. The plates were washed once more. Then, a two-component 3,3′,5,5′-tetramethylbenzidine substrate solution (Solarbio, Beijing, China) was used for color development in the dark for 30 min. Finally, color development was inhibited with a stop solution (Solarbio, Beijing, China), and the absorbance at a wavelength of 450/630 nm was measured.

For serum virus neutralization, serum samples were heat-inactivated at 56 °C for 30 min. A two-fold serial dilution of the serum was performed in a 96-well cell culture plate using DMEM medium, with three replicates for each sample. A total of 10^2^ TCID_50_ of influenza A virus A/California/07/2009 (H1N1) was added to each well containing the diluted serum and mixed thoroughly. the mixture was incubated at 37 °C for 2 h. Subsequently, the incubated mixture was transferred to a 96-well plate containing confluent MDCK cells and incubated again at 37 °C for 48 h. After incubation, cytopathic effects (CPE) were observed under a microscope and the results were validated using a hemagglutination assay (HA). The neutralizing antibody titer was defined as the highest serum dilution that completely prevented infection of MDCK cells.

### 2.9. Mouse Immunization and the Viral Challenge

The mice were divided into two main groups, HLA-A2/DR1 and HLA-A11/DR1, each comprising 10 mice. Each main group was then randomly divided into two subgroups, each including five mice. In the HLA-A2/DR1 main group, the first subgroup received two subcutaneous injections comprising a mixture of HLA-A*02:01-restricted peptides and adjuvant. In the HLA-A11/DR1 main group, the first subgroup received two subcutaneous injections of mixed HLA-A*11:01-restricted peptides and adjuvant. The second subgroups of the two main groups received two subcutaneous injections of Poly (I:C) (Invivogen, Toulouse, France). On day 14 after the booster immunization, the mice were anesthetized with isoflurane and then intranasally inoculated with 30 μL of 10^5^ TCID_50_/mL A/California/07/2009 (H1N1) influenza virus. On day 3 postinfection, the mice were euthanized, and their lungs were collected for influenza virus titration.

### 2.10. Titration of Influenza Virus in the Lungs of Mice

The mouse lung tissue samples obtained from the previous steps were homogenized in 1 mL DMEM using a cryogenic grinder, and the homogenate was stored at −80 °C. The virus titer was calculated based on the 50% tissue culture infective dose after serial dilution and infection of MDCK cells. Briefly, the mouse lung homogenates were serially diluted 10-fold with a serum-free medium containing 2 μg/mL of TPCK trypsin. Then, it was transferred to 96-well plates with a monolayer of MDCK cells and incubated at 37 °C under 5% CO_2_ for 2 h. After incubation, the virus solution was discarded, and 200 μL DMEM containing 0.2% BSA and 2 μg/mL TPCK trypsin was added to each well. Plates were further incubated for 48 h. Next, the cytopathic effect was observed under a microscope. The TCID_50_ value was calculated according to the Reed–Muench method and was expressed as TCID_50_/mL [21].

### 2.11. Statistical Analysis

Two-way analysis of variance was used for the statistical analysis of data from different mouse lines at two time points in the HI and ELISA antibody tests. The unpaired *t*-test was used to analyze data from the peptide immunization group and the adjuvant control group in the virus titer test. All statistical analyses were performed using GraphPad Prism (Version 8.0.2, La Jolla, CA, USA).

## 3. Results

### 3.1. Epitope Prediction and Screening

**Figure 1:**
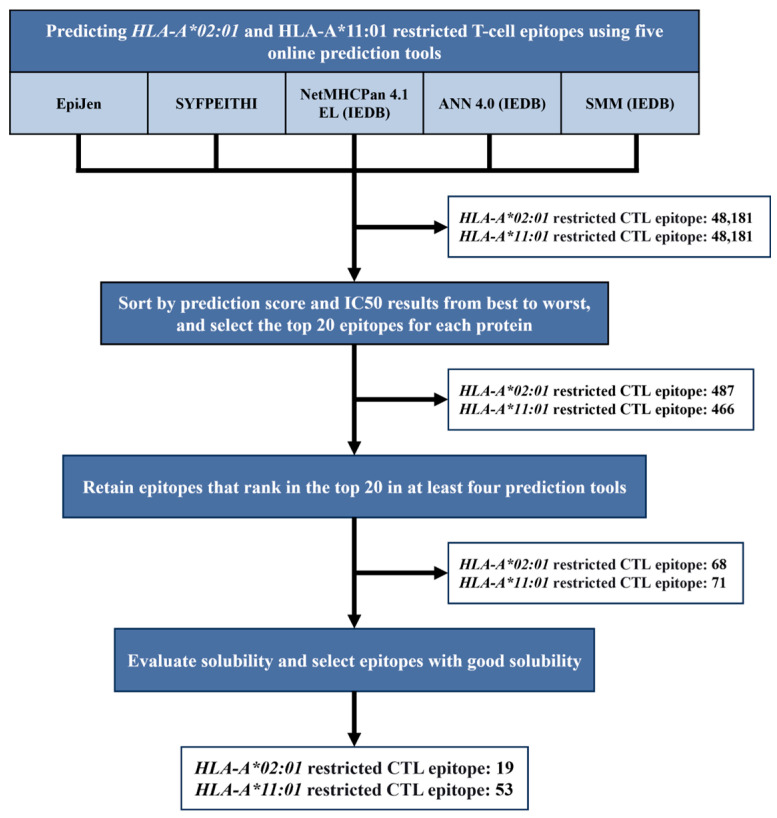
The flow chart and results of the epitope prediction and screening conducted in this study.

Figure 1 shows the prediction and screening strategy that was adopted and the results of the screening.

Immunoinformatics was utilized, and five online epitope prediction tools (EpiJen (Oxford, UK), SYFPEITHI (Heidelberg, Germany), NetMHCPan 4.1 EL (La Jolla, CA, USA), ANN 4.0 (La Jolla, CA, USA), and SMM (La Jolla, CA, USA)) were used to predict the HLA-A*02:01- and HLA-A*11:01-restricted T cell epitopes in the HA, NA, NP, PA, PB1, PB2, M1, M2, NS1, and NS2 proteins of the A/California/07/2009 (H1N1) influenza virus. The results of each prediction tool were ranked from the best to the worst based on scores and IC50 values, and the top 20 epitopes for each protein were selected, resulting in a total of 487 HLA-A*02:01-restricted epitopes and 466 HLA-A*11:01-restricted epitopes (Supplementary File S1). Then, we pooled the results from all prediction tools and retained the epitopes that were present in the top 20 in the results from at least four tools, thereby obtaining 68 HLA-A*02:01-restricted epitopes and 71 HLA-A*11:01-restricted epitopes. In addition, the hydrophilicity of these epitopes was assessed using a peptide solubility calculator (https://pepcalc.com/peptide-solubility-calculator.php, (accessed on 9 October 2024).). Ultimately, 19 water-soluble HLA-A*02:01-restricted epitope peptides (Table 1) and 53 water-soluble HLA-A*11:01-restricted epitope peptides were identified (Table 2). Among them, A2-8, A2-16, A2-17, and A2-18 have been confirmed to be HLA-A*02:01-restricted epitope peptides in previous studies [22,23]. A11-13 is considered an HLA-A*11:01-restricted epitope peptide in another study [24].

### 3.2. HLA-A2/DR1 and HLA-A11/DR1 Mice Can Generate Humoral Immune Responses After Influenza Virus Infection

**Figure 2:**
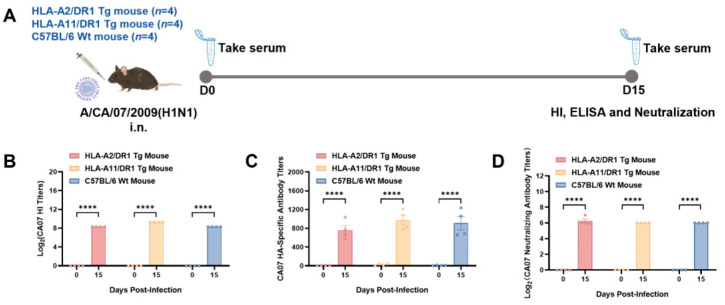
Antibody response in mice after intranasal infection. (**A**) Serum samples were collected from HLA-A2/DR1 transgenic mice (*n* = 4), HLA-A11/DR1 transgenic mice (*n* = 4), and C57BL/6 wild-type mice (*n* = 4) on days 0 and 15 after intranasal infection of the 10^1.5^ TCID_50_ A/CA/07/09 (H1N1) influenza virus. (**B**) Serum HI antibody titers on days 0 and 15 after intranasal infection. (**C**) Serum A/CA/07/09 (H1N1) influenza virus HA-specific antibody titers measured using ELISA on days 0 and 15 postinfection. (**D**) Serum A/CA/07/09 (H1N1) influenza virus neutralizing antibody titers on days 0 and 15 postinfection. **** p < 0.0001.

To assess the infection status of mice intranasally inoculated with the influenza virus, we followed the experimental design presented in Figure 2A and measured serum antibody levels on day 15 postinfection. The HI test, ELISA, and neutralization test results showed that on day 15 postinfection, HLA-A2/DR1 transgenic mice, HLA-A11/DR1 transgenic mice, and C57BL/6 mice had all produced antibodies against the A/California/07/2009 (H1N1) influenza virus (Figure 2B–D), and the serum antibodies had neutralizing activity. This indicates successful infection and the induction of humoral immunity.

### 3.3. HLA-A2/DR1 and HLA-A11/DR1 Mice Exhibited Cellular Immune Responses After Influenza Virus Infection

**Figure 3:**
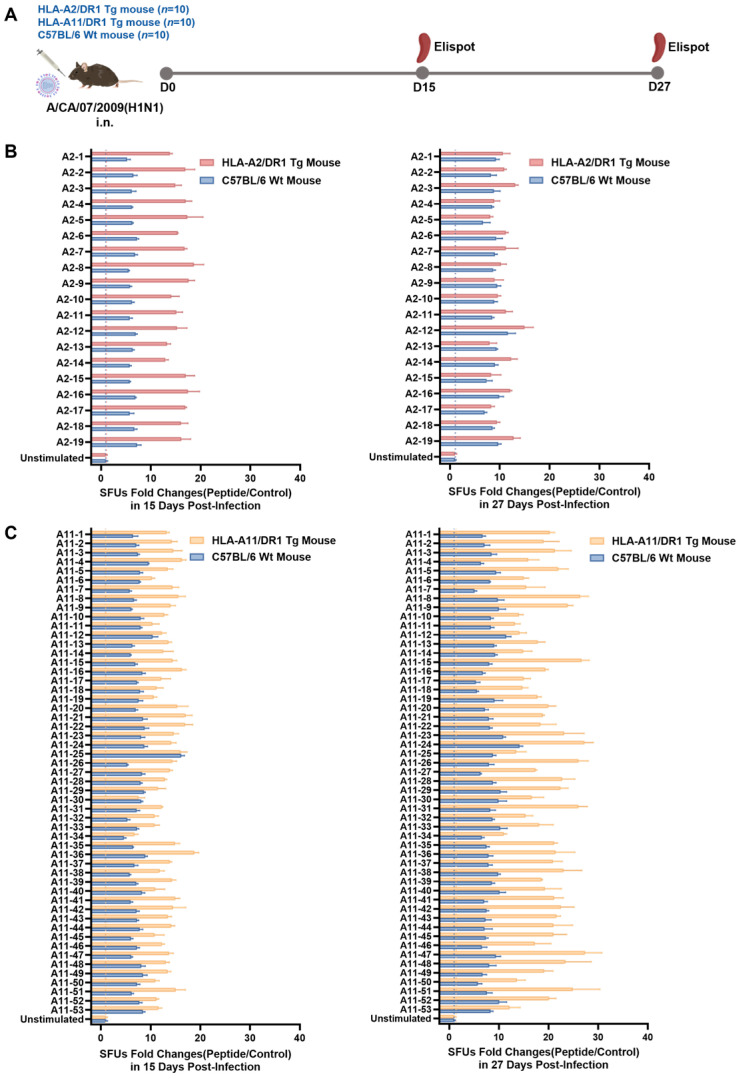
The predicted epitope peptides stimulated the production of specific IFN-γ in the spleen cells of infected mice. (**A**) HLA-A2/DR1 transgenic mice (*n* = 10), HLA-A11/DR1 transgenic mice (*n* = 10), and C57BL/6 wild-type mice (*n* = 10) were intranasally infected with 10^1.5^ TCID_50_ of A/CA/07/09 (H1N1) influenza virus. Their spleens were collected on days 15 and 27 postinfection for the ELISpot assay. (**B**) On day 15 (left) and day 27 (right) postinfection, the fold changes of IFN-γ spot forming units (SFUs) produced by HLA-A*02:01-restricted peptides stimulating HLA-A2/DR1 transgenic mice and C57BL/6 wild-type mice. The ordinate represents each peptide and cell control, whereas the abscissa represents the number of spots produced by 5 × 10^5^ transgenic or wild-type mouse spleen cells after stimulation with a single peptide, divided by the average number of spots in the corresponding cell control, to eliminate nonspecific spots produced by mouse spleen cells. The red dot line in the figure shows the fold change from irrelevant negative control peptide-stimulated HLA-A2/DR1 transgenic mice and the blue one from that stimulation in wild-type mice. (**C**) On day 15 (left) and day 27 (right) postinfection, the fold changes of IFN-γ SFUs produced by HLA-A*11:01-restricted peptides stimulating HLA-A11/DR1 transgenic mice and C57BL/6 wild-type mice. The coordinates were similar to those in Figure 3B. The yellow dot line in the figure shows the fold change from irrelevant negative control peptide-stimulated HLA-A2/DR1 transgenic mice, and the blue one from that stimulation in wild-type mice.

To validate the ability of HLA-A2/DR1 and HLA-A11/DR1 transgenic mice to screen for epitope peptides, we euthanized HLA-A2/DR1 transgenic mice, HLA-A11/DR1 transgenic mice, and C57BL/6 wild-type mice on days 15 and 27 postinfection to obtain splenocytes (Figure 3A). Each HLA-A*02:01-restricted epitope peptide from Table 1 was used to individually stimulate splenocytes from the wild-type and HLA-A2/DR1 transgenic mice. Further, each HLA-A*11:01-restricted epitope peptide from Table 2 was used to individually stimulate splenocytes from the wild-type and HLA-A11/DR1 transgenic mice. Additionally, the HLA-A*02:01-restricted Ebola virus epitope peptide LLADGLAKA (validated in prior studies) [25] and the HLA-A*11:01-restricted influenza epitope peptide QTNAMVTLR [26] were reciprocally used as irrelevant negative controls for the HLA-A*11:01 and HLA-A*02:01-restricted predicted influenza epitopes, respectively. Then, ELISpot assay was used to examine the ability of T cells to produce IFN-γ.

The HLA-A*02:01-restricted epitopes A2-8, A2-16, A2-17, and A2-18, which have been validated by other researchers [22,23], and the HLA-A*11:01-restricted epitope A11-13 [24] were used as positive peptides. Data from irrelevant negative control peptide stimulations were used as the nonspecific baseline. Results showed that all the HLA-A*02:01- and HLA-A*11:01-restricted epitope peptides successfully stimulated the splenocytes of the wild-type and transgenic mice to produce a specific cellular immune response on days 15 and 27, and the IFN-γ SFUs fold changes via stimulation were equivalent to or stronger than that of the positive peptides (Figure 3B,C). In addition, on day 15 postinfection, all 19 HLA-A*02:01-restricted epitope peptides stimulated the splenocytes of HLA-A2/DR1 transgenic mice more strongly than those of wild-type mice (Figure 3B). For the HLA-A*11:01-restricted epitope peptides, on day 15 postinfection, 39 of the peptides stimulated the splenocytes of the HLA-A11/DR1 transgenic mice more strongly than those of the wild-type mice. On day 27 postinfection, 40 of the peptides stimulated the splenocytes of the HLA-A11/DR1 transgenic mice more strongly than those of the wild-type mice (Figure 3C).

In summary, based on the ELISpot assay results, the HLA-restricted T cell epitope peptides that were predicted and selected successfully induced the T cells in the transgenic and wild-type mice to produce an immune response. Further, the transgenic mice reacted more strongly to most HLA-restricted epitopes than the wild-type mice.

### 3.4. Screening of Vaccine Candidate HLA-Restricted T Cell Epitope Peptides and Evaluation of Their Protective Efficacy

**Figure 4:**
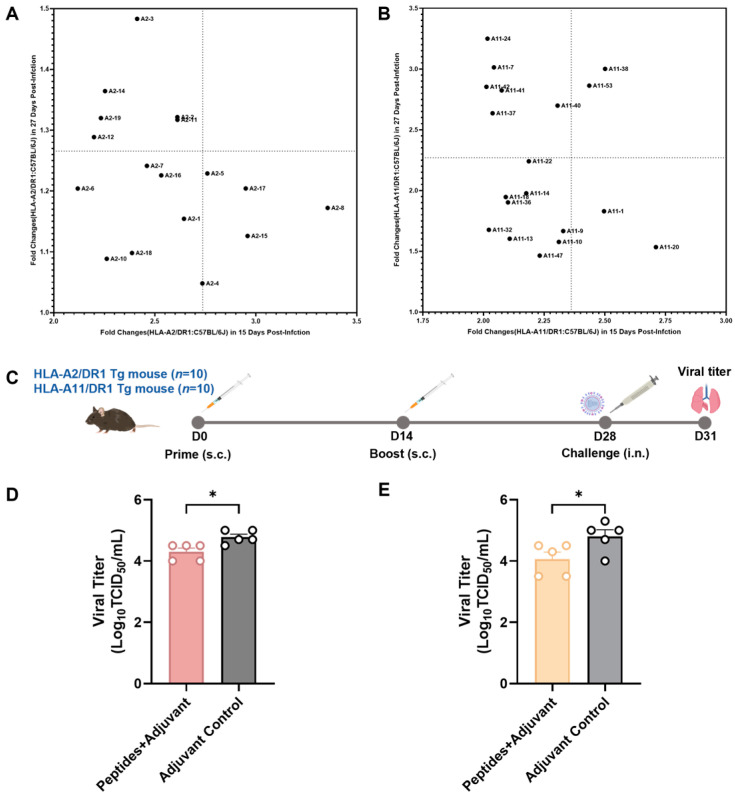
Screening of vaccine candidate peptides and evaluation of immune protective efficacy. (**A**) Fold changes of the HLA-A2/DR1 transgenic mice and C57BL/6 wild-type mice stimulated by HLA-A*02:01-restricted peptides on days 15 and 27 postinfection. (**B**) Fold changes of the HLA-A11/DR1 transgenic mice and C57BL/6 wild-type mice stimulated by HLA-A*11:01-restricted peptides on days 15 and 27 days postinfection. (**C**) The HLA-A2/DR1 transgenic mice and HLA-A11/DR1 transgenic mice were immunized twice with the selected candidate vaccine peptides and adjuvant. Meanwhile, the adjuvant control group was only injected with the adjuvant. Then, they were challenged with the influenza virus. To detect the virus titer, mouse lungs were collected on day 3 post-challenge. (**D**) The virus titer levels in the lungs of the HLA-A2/DR1 transgenic mice immunized with HLA-A*02:01-restricted mixed peptides and adjuvant were lower than those in the adjuvant control group. (**E**) The virus titer in the lungs of the HLA-A11/DR1 transgenic mice immunized with HLA-A*11:01-restricted mixed peptides and adjuvant were lower than those in the adjuvant control group. * p < 0.05.

To further investigate the protective effects of these epitope peptides as influenza vaccine candidate peptides, 10 peptides were selected each from the verified HLA-A*02:01- and HLA-A*11:01-restricted epitope peptides (Figure 4A,B) for immunization in the HLA-A2/DR1 transgenic and HLA-A11/DR1 mice. To obtain new fold change values, we initially divided the fold change values of the transgenic mice obtained from Figure 3B,C by the corresponding fold change values of the wild-type mice. These values can reflect the strong relationship of cellular immune responses between transgenic and wild-type mice after peptide stimulation. Larger values indicated that the cellular immune responses produced by peptide-stimulated transgenic mice were markedly stronger than those produced by wild-type mice. After removing the peptides with the fold changes less than or equal to 2 on day 15 postinfection, the fold change results on day 15 postinfection were taken as the X coordinates. Then, the ratio results on day 27 postinfection were considered as the Y coordinates. A reference line was drawn at the median of the fold change values on days 15 and 27 postinfection, respectively, thereby forming four quadrants. The first quadrant represented the peptides that ranked in the top 50% on day 15 postinfection. The second quadrant represented the peptides that ranked in the top 50% on days 15 and 27 days postinfection. The third quadrant represented the peptides that ranked in the bottom 50% at both time points. The fourth quadrant represented the peptides that ranked in the top 50% on day 27 postinfection. Then, the HLA-A*02:01-restricted epitope peptides and HLA-A*11:01-restricted epitope peptides located in quadrants 1, 2, and 4 were selected. The final screened HLA-A*02:01-restricted epitope peptides were A2-2, A2-3, A2-5, A2-8, A2-11, A2-12, A2-14, A2-15, A2-17, and A2-19. The HLA-A*11:01-restricted epitope peptides were A11-1, A11-7, A11-20, A11-24, A11-37, A11-38, A11-40, A11-41, A11-42, and A11-53.

The HLA-A2/DR1 transgenic and HLA-A11/DR1 transgenic mice were immunized according to the procedure shown in Figure 4C and were challenged with the influenza virus on day 14 after the final immunization. All mice were euthanized on day 3 post-challenge, and their lungs were collected to determine the virus titer.

The lungs of the HLA-A2/DR1 transgenic mice immunized with HLA-A*02:01-restricted mixed peptides and adjuvant had a significantly lower virus titer than those of the HLA-A2/DR1 transgenic mice injected with the adjuvant only (Figure 4D). Similarly, the lungs of the HLA-A11/DR1 transgenic mice immunized with HLA-A*11:01-restricted mixed peptides and adjuvant had a significantly lower virus titer than the lungs of HLA-A11/DR1 transgenic mice injected with the adjuvant only (Figure 4E). According to these findings, the candidate vaccine peptides screened using both types of transgenic mice exhibited protective effects against the influenza virus.

## 4. Discussion

Influenza virus remains a major global health and economic burden despite vaccination being the most effective preventive measure. Seasonal influenza persists due to rapid antigenic drift, enabling immune evasion through genomic evolution [4,9,27]. Thus, the influenza vaccines currently available in the market cannot provide long-term anti-infective protection, and immunity acquired after natural infection cannot prevent re-infection [28]. Although seasonal influenza viruses are highly variable during evolution, Heiny et al. analyzed influenza A viruses isolated from 10 consecutive years and identified 55 highly conserved 9-mer peptides on the PB2, PB1, PA, NP, and M1 proteins. These peptides are identical in over 80% of avian and human influenza A virus strains and contain T cell epitopes presented by HLA-I and II molecules [29]. Moreover, studies have shown that cross-reactive CTLs induced by conserved proteins of influenza A virus can offer heterologous immune protection among different influenza A strains [30,31,32]. Therefore, designing T cell epitope vaccines against influenza viruses is crucial.

Due to the rapid development of immunoinformatics, T cell epitopes with different HLA restrictions for multiple viruses could be predicted using online prediction tools to assist in designing T cell epitope vaccines [33,34]. However, these predicted epitopes should be validated via experiments. Experimental validation of predicted epitopes is essential but hindered by limited clinical samples and MHC complexity. Wild-type mice, lacking human HLA compatibility, are inadequate for HLA-restricted epitope screening. With improvements in gene editing technology, some transgenic mouse models that can simulate human immune responses have been developed and applied in human disease-related research [35,36,37]. In MHC-I humanized mice, HLA-I molecules can directly induce CTL responses, and these mice have been proven to be effective tools for research on infectious and autoimmune diseases, as well as for vaccine development and testing [38]. HLA-II humanized mice have also been used to study various human immune-related diseases [39].

In this study, we employed HLA-A2/DR1 and HLA-A11/DR1 double-transgenic mice, engineered to express human HLA-A*02:01, HLA-A*11:01, and HLA-DRB1*01:01 while lacking endogenous MHC genes. Studies have shown that HLA-A02/DR1 transgenic mice and HLA-A11/DR1 transgenic mice immunized with recombinant HBV vaccine develop adaptive immune responses consistent with human immune features, including serum-specific antibodies and HLA-A*02:01- and HLA-A*11:01-restricted CTL responses [18,40]. We intranasally infected the two transgenic mouse strains and wild-type mice with the A/California/07/2009 (H1N1) influenza virus. Results showed the presence of antibodies in the serum. This finding indicated that all three mice strains were susceptible to viral infection and that they exhibited humoral immune responses. Simultaneously, the HLA-A*02:01- and HLA-A*11:01-restricted T cell epitope peptides predicted and selected using immunoinformatics were used to stimulate the HLA-A2/DR1 transgenic mice, HLA-A11/DR1 transgenic mice, and wild-type mice after viral infection. Results showed that all epitope peptides, including those previously reported as positive, could induce cellular immune responses in mice. Moreover, after eliminating the baseline IFN-γ produced by the splenocytes of the mice, the IFN-γ levels of the transgenic mice were higher than those of the wild-type mice. These results indicate that HLA-A2/DR1 and HLA-A11/DR1 transgenic mice can be infected by the influenza virus. However, they can produce both humoral and cellular immune responses, with their cellular immune response being stronger than that of C57BL/6 wild-type mice. Subsequently, the epitope peptides that had been validated in mice were re-screened, and 10 peptides each for HLA-A*02:01 and HLA-A*11:01 were selected to immunize the transgenic mice. The mice were subjected to viral challenge after two immunizations to assess the protective effects of these peptides. Results showed that the immunized mice exhibited significantly lower viral replication than the adjuvant control group. This confirmed that HLA-A2/DR1 and HLA-A11/DR1 transgenic mice could be directly used for the screening of the T cell epitope peptides of the influenza virus.

HLA is highly polymorphic, and the catalog of common and documented HLA alleles includes 1122 alleles for the HLA-A, -B, -C, -DRB1, -DRB3/4/5, -DQA1, -DQB1, -DPA1, and -DPB1 loci [41]. Our research has revealed that HLA-A2/DR1 and HLA-A11/DR1 transgenic mice are effective for the screening of HLA-A*02:01- and HLA-A*11:01-restricted T cell epitopes. Therefore, if more humanized MHC mouse models with other MHC alleles are established, a wider range of human alleles can be covered, and T cell epitope vaccines that provide broader protection can be designed.

In fact, some T cell epitope-based influenza vaccines have entered the clinical trial stage. Clinical trials indicate that FLU-v, a novel influenza vaccine targeting conserved T cell epitopes in NP, M1, and M2, is safe and can induce vaccine-specific cellular immunity [42]. Human studies have shown that the vaccine FP-01.1, which incorporates multiple CD4^+^ and CD8^+^ T cell epitopes, induces a robust antiviral T cell response in a high proportion of tested participants. Moreover, it has acceptable safety and tolerance [43]. Targeting conserved regions of the influenza virus that can induce CD8^+^ T cell responses, as well as B cell and antibody responses, may be one of the avenues for developing a universal next-generation influenza vaccine [44,45]. The epitope peptides selected using humanized MHC mice can effectively inhibit viral replication. However, the immunogenicity of the peptides is relatively weak, and peptide immunization does not induce a strong humoral immune response. Combining epitopes with protective antigens could potentially enhance the immune response, warranting further investigation. In recent years, many new T cell-based influenza vaccines have been in various stages of clinical development. Examples include the viral vector vaccines ChAdOx1NP+M1 [46] and MVA-NP+M1 [47], the recombinant protein vaccines M-001 [48] and OVX836 [49], etc. All of them can induce CD8^+^T cell responses and show good protective effects. Moreover, a study has demonstrated that the combination of seasonal influenza vaccines and MVA-NP+M1 can significantly boost T cell immune responses to influenza internal proteins [50].

The current study offers a strategy that allows for the direct use of humanized MHC mice in epitope screening without the necessity of clinical samples. Humanized MHC mice can aid in the screening of influenza T cell epitopes and be used in the epitope screening of other viruses. For instance, Nascimento et al. used six HLA transgenic mouse strains, including HLA-A*02:01, to validate over 90 DENV3 T cell epitopes [51]. Miranda-Katz et al. utilized HLA-B*07:02 transgenic mice to identify HLA-B*07:02-restricted CD8^+^T cell epitopes from a human metapneumovirus epitope library [52]. In studies of deadly pathogens, Bounds et al. screened T cell epitopes for the Ebola virus, Sudan virus, and Venezuelan equine encephalitis virus in HLA transgenic mice [53]. In addition, Gardyan et al. pioneered the use of HLA-DRB1*03:01 and HLA-DRB1*04:01 transgenic mice to identify HLA class II-restricted CD4^+^T cell epitopes from the breast cancer antigen NY-BR-1, offering new targets for cancer immunotherapy [54]. Therefore, if a novel or emerging infectious disease arises, this strategy can be used in the epitope screening of the pathogen before the spread of outbreaks and for designing vaccines, thereby providing strong support for the control of the epidemic.

## 5. Conclusions

In this study, we predicted and screened the HLA-restricted T cell epitope peptides of the IAV and validated them using HLA-A2/DR1 and HLA-A11/DR1 transgenic mice. All the peptides that were selected could induce cellular immune responses in mice. Based on the results of the viral challenge, the validated peptides provided protection to the mice. These findings indicated that HLA-A2/DR1 and HLA-A11/DR1 transgenic mice can be directly used for the screening and validation of HLA-restricted T cell epitope peptides of the influenza virus. Further, it can be a feasible method for epitope screening and vaccine designing before the spread of other emerging and sudden infectious diseases.

## Figures and Tables

**Table 1:** HLA-A*02:01-restricted epitope peptides.

**Name**	**Peptide**	**Protein**	**Starting Position**	**Ending Position**	**Length**	**HLA Restriction**
A2-1	TVLEKNVTV	HA	35	43	9	HLA-A*02:01
A2-2	AIDEITNKV	HA	388	396	9	HLA-A*02:01
A2-3	KLNREEIDGV	HA	511	520	10	HLA-A*02:01
A2-4	RMGTVTTEA	M1	134	142	9	HLA-A*02:01
A2-5	AIYSKDNSV	NA	98	106	9	HLA-A*02:01
A2-6	KLSDYDGRL	NP	48	56	9	HLA-A*02:01
A2-7	GMDPRMCSL	NP	158	166	9	HLA-A*02:01
A2-8	TMDSNTLEL	NP	373	381	9	HLA-A*02:01
A2-9	YLSDMTLEEM	NS1	89	98	10	HLA-A*02:01
A2-10	GMVTRFESL	NS2	17	25	9	HLA-A*02:01
A2-11	SLGETVMRM	NS2	31	39	9	HLA-A*02:01
A2-12	VMRMGDLHYL	NS2	36	45	10	HLA-A*02:01
A2-13	QLGQKFEEI	NS2	55	63	9	HLA-A*02:01
A2-14	WLIEEMRHRL	NS2	65	74	10	HLA-A*02:01
A2-15	QLLLEVEQEI	NS2	91	100	10	HLA-A*02:01
A2-16	ALLKHRFEI	PA	70	78	9	HLA-A*02:01
A2-17	SMIEAESSV	PA	594	602	9	HLA-A*02:01
A2-18	RLIDFLKDV	PB1	162	170	9	HLA-A*02:01
A2-19	SLLEMCHST	PB2	279	287	9	HLA-A*02:01

**Table 2:** HLA-A*11:01-restricted epitope peptides.

**Name**	**Peptide**	**Protein**	**Starting Position**	**Ending Position**	**Length**	**HLA Restriction**
A11-1	GVAPLHLGK	HA	63	71	9	HLA-A*11:01
A11-2	SSWPNHDSNK	HA	138	147	10	HLA-A*11:01
A11-3	HAGAKSFYK	HA	155	163	9	HLA-A*11:01
A11-4	FVGSSRYSK	HA	217	225	9	HLA-A*11:01
A11-5	NIHPITIGK	HA	311	319	9	HLA-A*11:01
A11-6	NTQFTAVGK	HA	404	412	9	HLA-A*11:01
A11-7	RSQLKNNAK	HA	467	475	9	HLA-A*11:01
A11-8	RLESVFAGK	M1	27	35	9	HLA-A*11:01
A11-9	FTLTVPSER	M1	64	72	9	HLA-A*11:01
A11-10	TVPSERGLQR	M1	67	76	10	HLA-A*11:01
A11-11	NMDRAVKLYK	M1	92	101	10	HLA-A*11:01
A11-12	AVKLYKKLK	M1	96	104	9	HLA-A*11:01
A11-13	RLFFKCIYRR	M2	44	53	10	HLA-A*11:01
A11-14	STEGVPESMR	M2	63	72	10	HLA-A*11:01
A11-15	LTQGALLNDK	NA	134	143	10	HLA-A*11:01
A11-16	GTIKDRSPYR	NA	147	156	10	HLA-A*11:01
A11-17	KSWRNNILR	NA	217	225	9	HLA-A*11:01
A11-18	ASYKIFRIEK	NA	251	260	10	HLA-A*11:01
A11-19	SSNGANGVK	NA	339	347	9	HLA-A*11:01
A11-20	SAFDERRNK	NP	69	77	9	HLA-A*11:01
A11-21	SLMQGSTLPR	NP	165	174	10	HLA-A*11:01
A11-22	RSGAAGAAVK	NP	175	184	10	HLA-A*11:01
A11-23	RVSSFIRGKK	NP	342	351	10	HLA-A*11:01
A11-24	SVQPTFSVQR	NP	407	416	10	HLA-A*11:01
A11-25	SVQRNLPFER	NP	413	422	10	HLA-A*11:01
A11-26	GVFELSDEK	NP	462	470	9	HLA-A*11:01
A11-27	KQIVEWILK	NS1	62	70	9	HLA-A*11:01
A11-28	KIIGPLCVR	NS1	110	118	9	HLA-A*11:01
A11-29	RLDQAIMEK	NS1	118	126	9	HLA-A*11:01
A11-30	AIMEKNIVLK	NS1	122	131	10	HLA-A*11:01
A11-31	RLETLILLR	NS1	140	148	9	HLA-A*11:01
A11-32	MVTRFESLK	NS2	18	26	9	HLA-A*11:01
A11-33	DSLGETVMR	NS2	30	38	9	HLA-A*11:01
A11-34	TVMRMGDLH	NS2	35	43	9	HLA-A*11:01
A11-35	SICNTTGVEK	PA	93	102	10	HLA-A*11:01
A11-36	ASWVQNEFNK	PA	404	413	10	HLA-A*11:01
A11-37	RTNGTSKIK	PA	566	574	9	HLA-A*11:01
A11-38	SVKEKDMTK	PA	601	609	9	HLA-A*11:01
A11-39	MTKEFFENK	PA	607	615	9	HLA-A*11:01
A11-40	KVCRTLLAK	PA	635	643	9	HLA-A*11:01
A11-41	NSFLTHALK	PA	708	716	9	HLA-A*11:01
A11-42	QTYDWTLNR	PB1	127	135	9	HLA-A*11:01
A11-43	MVTQRTIGK	PB1	199	207	9	HLA-A*11:01
A11-44	SILNLGQKK	PB1	422	430	9	HLA-A*11:01
A11-45	ATTHSWIPK	PB1	661	669	9	HLA-A*11:01
A11-46	SSMVEAMVSR	PB1	712	721	10	HLA-A*11:01
A11-47	TTVDHMAIIK	PB2	23	32	10	HLA-A*11:01
A11-48	TTSTVHYPK	PB2	105	113	9	HLA-A*11:01
A11-49	STVHYPKVYK	PB2	107	116	10	HLA-A*11:01
A11-50	KVYKTYFEK	PB2	113	121	9	HLA-A*11:01
A11-51	GTFGPVHFR	PB2	128	136	9	HLA-A*11:01
A11-52	RTSGSSVKK	PB2	332	340	9	HLA-A*11:01
A11-53	SINELSNLAK	PB2	709	718	10	HLA-A*11:01

## Data Availability

The data presented in this paper are available on request from the corresponding author.

## Supplementary Materials

The following supporting information can be downloaded at: https://www.mdpi.com/article/10.3390/vaccines13030331/s1, File S1: All 487 *HLA-A*02:01*-restricted and 466 *HLA-A*11:01*-restricted epitopes.
